# The private sector and universal health coverage

**DOI:** 10.2471/BLT.18.225540

**Published:** 2019-04-01

**Authors:** David Clarke, Shana Doerr, Mostafa Hunter, Gerard Schmets, Agnes Soucat, Aurelie Paviza

**Affiliations:** a Department of Health Systems Governance and Financing, World Health Organization, avenue Appia 20, 1211 Geneva 27, Switzerland.; b UHC2030, World Health Organization, Geneva, Switzerland.; c Independent consultant, 45 Shehab street, Giza, Egypt.

The sustainable development goals (SDGs) of *Transforming our world, the 2030 agenda for sustainable development*,^1^ and specifically SDG 17, call for cooperation, collaboration and partnership between government, civil society and businesses. To reach the agenda’s objectives, the international community needs to find ways to effectively harness the public and private sectors. The SDGs are integrated and indivisible, with progress in one area dependent upon progress in others. Both the private and public sector are needed to meet the health-related SDG 3, including the target of universal health coverage (UHC) and related goals such as SDG 8 (decent work and economic growth) and SDG 9 (industry, innovation and infrastructure).

In the health area, the private sector refers to all non-state actors involved in health: profit and not-for-profit, formal and informal, domestic and international. Almost all countries have mixed health systems, with goods and services provided by the public and private sector, and health consumers requesting these services from both sectors. Therefore, efforts towards UHC cannot ignore the private sector. The private sector’s involvement in health systems is significant in scale and scope and includes the provision of health-related services, medicines and medical products, financial products, training for the health workforce, information technology, infrastructure and support services.

The private sector’s role in health care is growing because it offers solutions to many challenges that have a negative impact on health systems including: health fiscal space constraints, increases in disease burden, particularly in relation to noncommunicable diseases, demographic shifts including ageing, population displacement and political and economic instability. 

The private sector has contributed to addressing these challenges. One example is its engagement with the World Health Organization’s (WHO) End TB Strategy.^2^ Many governments and donors promote the private sector as a solution because it is perceived as offering access to greater service capacity, more managerial expertise, higher quality of services, and technology and innovation, as well as investment and funding.^3^ However, major gaps in our knowledge about the benefits of private care provision remain. In particular, the international community, including WHO, lacks an evidence base from which to develop guidance about the types of services and activities where the private sector might have a comparative advantage over the public sector.

Private sector engagement also involves risks. Governments in low- and middle-income countries face major challenges with the private sector because their existing governance and regulatory arrangements are not designed to effectively manage and coordinate mixed health systems. One of the challenges relates to the for-profit private sector. In many countries, this sector has not been properly managed or regulated, resulting in behaviours that threaten the UHC objectives of equity and quality.^4^ Such behaviours include the abuse of market power (market skimming monopolistic behaviour and predatory pricing), unresolved conflicts of interest and regulatory capture. Another challenge is how to harness the efforts of the not-for-profit private sector to help meeting the health objectives of governments. While the business model of many not-for-profit private providers aligns well with UHC, governments often have incomplete information about the not-for-profit providers and lack the governance tools to help align the activities of these providers with national systems and priorities.^5^

Many countries do not have an explicit government policy position on the role of the private health sector, nor concrete plans to implement public policy on the private sector.^6^ To date, few countries have engaged in structured debate or multistakeholder dialogue about the role of the private sector and UHC. As a result, there is often no consensus among domestic stakeholders, including health systems users and civil society groups, about the role the private sector should play in health.^6^

Therefore, a public policy vacuum exists regarding the private sector and UHC. In this vacuum, the private sector could pursue its own objectives, which may or may not be closely aligned to UHC. This situation is related to private-sector interventions that are often supply, rather than demands driven and such interventions are inconsistent with the Paris Declaration on Aid Effectiveness.^7^ Hence, demand driven interventions determined with the participation of government and local stakeholders, including patient groups, are needed, because such interventions are more aligned and coordinated with national priorities and subject to domestic accountability. 

Ignoring the role of the private sector in national efforts towards UHC is not an option. Here we suggest the following approach to managing, and where appropriate, engaging the private sector as part of efforts to achieve UHC.

First, governments should take the lead and formulate domestic health goals and priorities. Based on these goals, governments can then formulate public policies about the role of the private sector for UHC, orienting the health systems towards achieving UHC. Second, as the private sector is heterogeneous, context-specific policy approaches are required to align the work of the private sector with the goal of achieving UHC. Therefore, the choice and implementation of public-private UHC policies need to be informed by an understanding of the different private sector actors that operate in a country. The attributes of these actors include whether they are for-profit or not-for-profit, their social intentions, whether they have domestic or foreign affiliations, their social and ethical behaviour, and their willingness to support the government’s health policies. After mapping the different private sector actors in a health system, governments should engage in multistakeholder dialogues to establish their policy on the private sector and UHC. Multistakeholder involvement is important because it can uncover risks and conflict of interests as well as strengthen accountability within the health system.^8^ Third, is implementation of the government’s policy on the role of the private sector for UHC, with a mix of legal and financial regulatory tools to manage the private sector and steer efforts towards achieving UHC.

WHO plays a key role in supporting Member States with this critical area of work. The organization works to strengthen the governments’ capacity to make informed decisions about the role of the private sector. For this purpose, WHO has developed a new conceptual framework and a decision-making model^6^ to help guide countries through the approaches recommended above.

High-income countries with substantial private sector involvement in health care already have a set of institutionalized policy instruments for managing the private sector, including regulatory mechanisms for licensing, certification and accreditation of health workers, medical products, services and facilities. These countries also have regulatory governance arrangements with a workforce of trained officials to help and administer these instruments.^6^ In middle- and low-income countries, the situation is more challenging. Considering the rapid development of the private sector, these countries need to develop and implement context-specific and appropriate policy and regulatory instruments and a workforce to implement them. At the same time, countries need to adopt accountability mechanisms to ensure that any public-private partnership serves the health of the population and the goal of UHC.

As the coordinating authority on health within the United Nations (UN) system, WHO is widely perceived as a neutral organization with no vested interest to support private health-care provision. WHO can therefore ensure the topic is addressed while safeguarding core health values of equity, quality and financial protection.

WHO is actively working to support Member States to develop policy on the private sector and UHC, strengthen the Member States’ capacity to make informed decisions, and develop and implement suitable regulatory and financial tools for managing the private sector and public–private partnerships. Moreover, international health partnerships, such as UHC2030^9^ are providing a platform for WHO, the World Bank, civil society organizations and other partners to engage in dialogue with the private sector regarding the role of the private sector in reaching UHC.

With the UN High-level meeting on UHC scheduled for September 2019, an open debate on the role of the private sector in UHC is timely. WHO, in partnership with UHC2030, intends to promote global and country multistakeholder dialogue on this topic, giving countries the lead in efforts towards UHC.
